# Characterisation of Particulate Matter Emitted from Cofiring of Lignite and Agricultural Residues in a Fixed-Bed Combustor

**DOI:** 10.1100/2012/702451

**Published:** 2012-05-02

**Authors:** Nattasut Mantananont, Savitri Garivait, Suthum Patumsawad

**Affiliations:** ^1^The Joint Graduate School of Energy and Environment, King Mongkut's University of Technology Thonburi, Bangkok 10140, Thailand; ^2^Department of Mechanical Engineering, King Mongkut's University of Technology North Bangkok, Bangkok 10800, Thailand

## Abstract

This study is focused on the emission of fixed bed combustor batch operated. Real-time analyser ELPI (electrical low-pressure impactor) system was used to size-segregated particulate matter emission ranging from 40 nm to 10 *μ*m. The results show that total number concentration were 3.4 × 10^3^, 1.6 × 10^4^, and 1.5 × 10^5^ particles/cm^3^ · kg_fuel_, while total mass of particles were 12.2, 8.0, and 6.5 mg/Nm^3^ · kg_fuel_ for combustion of lignite, rice husk and bagasse, respectively. But it can be noticed that cofiring released more particulate matter. Meanwhile it was found that the effect of ratio of over-fired air to total air supply is more pronounced, since decrease in this ratio, the amount of particles are decreased significantly. For particle size distribution, it can be observed that submicron-sized particles dominate and the most prevailing size is in the range: 50 nm <*D*_*p*_ < 100 nm, for lignite and agricultural residues. However, during cofiring of fuel mixture at 70% rice husk mass concentration, it is found that there are two major fractions of particle size; 40 nm <*D*_*p*_ < 70 nm and 0.2 *μ*m <*D*_*p*_ < 0.5 *μ*m. The analysis of particle morphology showed that the isolate shape of submicron particle produced during lignite combustion is characterised by different geometries such as round, capsule, rod, flake-like, whereas the spherical shape is obtained with combustion of rice husk.

## 1. Introduction

Airborne particulate matter (PM) is one of the major pollutants affecting negatively the atmospheric environment, combustion system, and human health. For its impact on atmospheric environment, it is known that sub-micron-sized particle (e.g., 0.1–1 *μ*m) whether in form of solid or droplet plays a role to decrease visibility [1]. In problematic of combustion system [2], there were reports that serious corrosion problems were found in the cooler part of the flue gas path. From SEM-EDS analysis, it indicated that the corroded tube was covered with oxide layer having rich of Fe, K, Cl, Si, and S, which these elements mostly contain in submicron particle.

The major fuel for energy production in Thailand is lignite; however, its amount is limited in a long term. As agricultural countries, Thailand produces large amount of agricultural residues such as rice husk, and bagasse [3]. In the light of these, the energy production by co-firing lignite/residues becomes a promising option. PM emission during cocombustion of coal/biomass/wastes has broadly been investigated [4–10], but they are mostly processed by densification and burnt as pellet or briquette. Meanwhile, in Thailand, study of emission from cocombustion of domestic lignite, biomass, and waste has been investigated [11] but they focused only on gaseous emission and combustion efficiency associated with combustion condition regardless to the measurement of particulate matter. In fact, the characteristics of particulate emitted either from combustion of Thai lignite, rice husk, and bagasse or from cofiring of Thai lignite/rice husk have not been investigated in Thailand up to now.

This study is, therefore, focused on the PM emission from lab-scale fixed bed combustor batch operated. The point of study includes total number/mass concentration of PM, and determination of particle morphology. The effect of the fuel mixture and the ratio of overfired air to total air supply (OFA/TA) on PM characteristics has also been addressed.

## 2. Experimental Setup

### 2.1. Fuel Preparation and Properties

**Figure 1:**
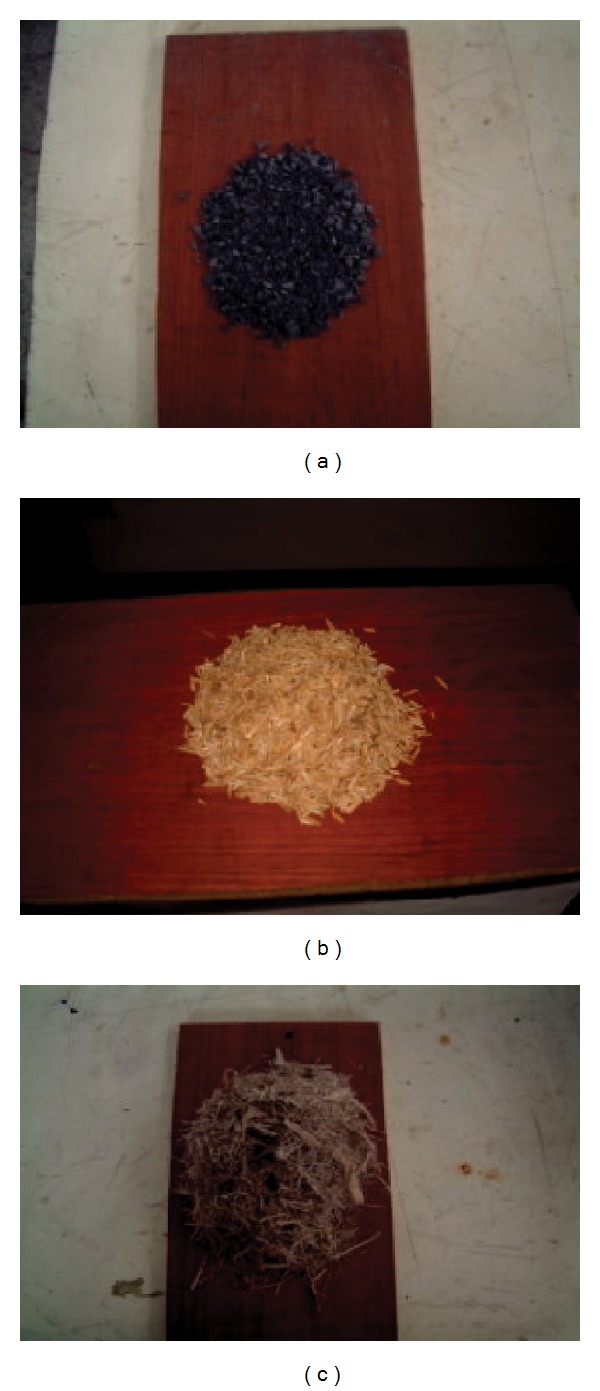
fuel physical (a) lignite, (b) rice husk, and (c) bagasse.

In this study, domestic lignite and two agricultural residues; rice husk and bagasse have been selected. Their physical are depicted by Figure 1. Lignite was supplied by the Electricity Utility in Thailand and was crushed and sieved to 3–5 mm in diameter range. Rice husk and bagasse were received from rice mills and sugar cane factory, respectively, and used in as-received characters, as shown in Figure 1. Since bagasse physical is inhomogeneous in size/shape (e.g., short-long line, thinner-thicker shape, or powder portion) including low density (60 kg/m^3^) in comparison with lignite, making difficult to well mixing with lignite, so combustion of bagasse alone is present in this paper. However, rice husk seemed better to mix with lignite; hence cofiring of lignite and rice husk can be tested.

For cofiring of lignite and rice husk, they were mixed together before loading to reactor. The fuel mixtures of lignite and rice husk are 30/70 and 60/40 by mass concentration. Burning 100% of lignite, rice husk and bagasse are performed as baseline data. Fuel properties are shown in Table 1.

### 2.2. Experimental Rig

**Figure 2:**
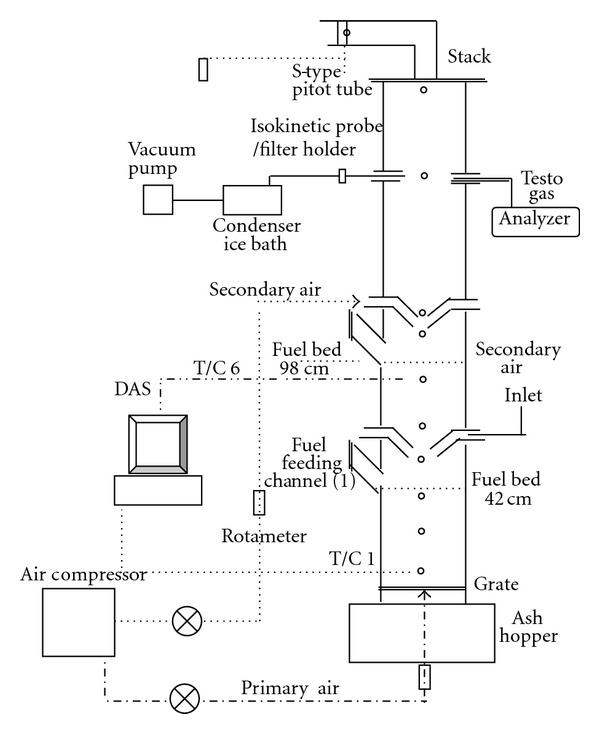
Fixed-bed combustor and particulate sampling system.

The experiments were conducted in a lab-scale fixed bed combustion system which is a vertical cylindrical chamber of 120 mm internal diameter, 2680 mm height, insulating with 45 mm of refractory material, and 20 mm of rock wool. The grate is located at the bottom. Eleven thermocouples (types K chromel-alumel) were used to measure the temperature along the reactor including combustion zone and freeboard. Air supply was divided into two parts, named as overfired and underfired air. Underfired air was fed beneath the grate while overfired air was put at 840 mm height above the grate. The ratio of overfired air to total air (OFA/TA) was increased from 0 to 0.3.  Figure 2 shows the schematics of experimental setup.

### 2.3. Particulate Sampling and Analysis

Real-time analyser: ELPI (electrical low-pressure impactor) was used to size-segregated PM emission ranging from 40 nm to 10 *μ*m. Sampling probe was inserted in fixed-bed reactor located at 2680 mm above the grate. Twenty-five millimetre of diameter of Thirteen-Teflon filters without greasing was used to sampling particulate per one case. Particle sized larger than 10 *μ*m was trapped at 13th stage of ELPI and only particle sized below 10 *μ*m was allowed through size-segregation 1st–12th stages of ELPI. The sampling rate was fixed constantly at 10 L/min entire the sampling time. Number of particle is a function of measured current and mass concentration of particle was calculated based on 1 g/cm^3^ of particle density. Total number and total mass of particle were obtained by integration of value in each stage (i.e., 1st–12th). 

SEM (scanning electron microscopy) model: JSM-6301F was used to study the particle morphology. The analysis of particle morphology covered the significant mode of particulate, especially submicron particle, nucleation mode (*D*_*p*_; 70–100 nm), or 2nd stage of filter, accumulation mode (*D*_*p*_; 0.2–0.31 *μ*m) or 4th stage including supermicron particle (*D*_*p*_; 3–5 *μ*m) or 10th stage. 

## 3. Results and Discussion

### 3.1. Total Number/Mass Concentration of Particle Emitted from Combustion

The results are shown in Table 2. The total number of particle emitted from combustion of lignite, rice husk, and bagasse are 3.4 × 10^3^, 1.6 × 10^4^, and 1.51 × 10^5^ particles/cm^3^ · kg_fuel_, respectively, while total mass of particles are 12.2, 8.0, and 6.5 mg/Nm^3^ · kg_fuel_. These results indicate roughly that combustion of low bulk density fuel may be one of the causes to generate higher the emitted particle (compared to lignite). 

However, comparison with 1.8 × 10^13^, 1 × 10^13^, 1.7 × 10^13^ particle/kg released from combustion in self-built burning stove of wheat straw, corn straw, and rice straw, respectively [13]. It seems that the low of density fuel (i.e., rice husk or bagasse) may not be a priority concerned with high emission of PM but the combustion technology or operating condition seems more importance. 

Other interesting is that bagasse and rice husk have higher volatile yield than coal, therefore, the main combustion process is marked by devolatilisation rate of fuel and homogeneous (gas-phase) reaction dominated, which later favours particle formation via gas-to-solid pathway (e.g., condensation). According to this phenomenon, this could be observed from the reverse relationships between particle number and particle mass concentration. For instance, most prevailing size of particle of bagasse combustion is at *d*_*p*_ 70 nm of 80% cumulative of total number of particle. Because this high content of submicron particles is less significant to contribute the overall mass loading, thus low mass concentration does. In addition, low mass of emitted particle also indicates the lower particle density of residues. 

### 3.2. Total Number/Mass Concentration of Particle Emitted from Cofiring of Lignite and Rice Husk

Cofiring of lignite and rice husk was performed under various mass fraction and ratio of overfired air to total air and results are shown in Table 3. 

It can be seen that cofired lignite and rice husk result in increase of both particle number and mass concentration compared to burning of either lignite or rice husk. This synergy effect could be from the difference in fuel properties and physical which needed further investigation and analysis. While mass fraction concentration has affected to PM emission, an increase in lignite mass concentration leads to decrease in PM emission. However, total number/mass concentration of particle is decreased dramatically at overfired air to total air ratio of 0.1. The result of particle number of fuel mixture (8.7 × 10^3^) is in between those of lignite and rice husk (3.4 × 10^3^ and 1.6 × 10^4^) but mass concentration is much lower. This could be said that PM emission at this condition probably is very fine particle. 

### 3.3. Particle Size Distribution (PSD) of Combustion

As from the results, it was found that the most prevailing particle size was in the range of 50 nm to 100 nm for combustion of lignite and bagasse. It was accounted to 60 and 80% of total particle for lignite and bagasse, respectively. Meanwhile, for rice husk combustion, it was obviously seen that there were two groups of particle size range; 50–100 nm and 0.5–1.0 *μ*m. This could be inferred that ultrafine or fresh particle was collided and agglomerated to form fine particle. 

### 3.4. Particle Size Distribution (PSD) of Cofiring of Lignite and Rice Husk

The results from cofiring lignite with rice husk show the same effect as burning of either lignite or agricultural residues. The major fraction of particle size is 40–70 nm but the number of particle is higher. However, the increase in rice husk mass fraction, 40 to 70%, leads to release larger particle size. At 70% rice husk mass fraction, there are two modes of particle size range; 40–70 nm and 0.2–0.5 *μ*m, which are agreed with the results of rice husk combustion. 

### 3.5. Particle Morphology

**Figure 3:**
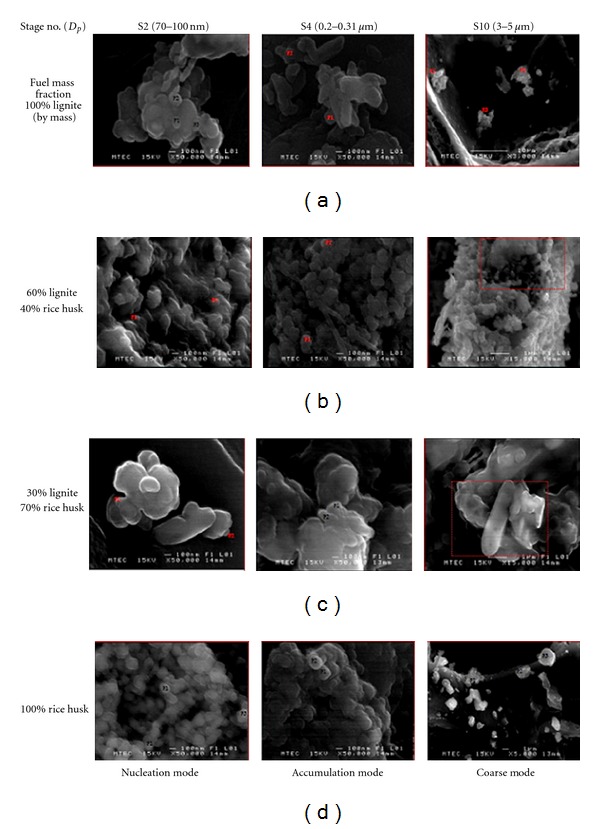
Particle morphologies.

SEM was used to investigate particle morphology. Particle shape derived from combustion of lignite, rice husk, and cofiring of lignite/rice husk are illustrated by Figures 3(a)–3(d). It can be seen from Figure 3(a) that the isolate shape of submicron particle produced during lignite combustion is characterised by different geometries such as round, capsule, rod, flake-like, whereas the spherical shape is obtained from rice husk combustion (see Figure 3(d)). 

For cofiring mode, Figure 3(c) (left and middle) shows that co-firing of high mass fraction of rice husk (70%) enables to modify structure of submicron particle from “small-roundly shaped” to “large-amorphously shaped,” in comparison to rice husk burning case, which finally results in increasing of the average diameter of particle. 

## 4. Conclusion

Characterisation of particulate matter emitted from firing and cofiring of lignite and agricultural residues, rice husk and bagasse, has been investigated in fixed-bed combustor batch operated. Parameters concerned in this study are comprised of total number/total mass concentration and particle morphology. It can be summarised the results as follows.

(1) Total number concentration was 3.4 × 10^3^, 1.6 × 10^4^, and 1.5 × 10^5^ particles/cm^3^ · kg_fuel_, while total mass of particles was 12.2, 8.0 and 6.5 mg/Nm^3^ · kg_fuel_ for combustion of lignite, rice husk, and bagasse, respectively.

(2) In cofiring of lignite and rice husk, the results show synergy effect which released particulate matter is higher than burning either lignite and rice husk. The increase in rice husk mass fraction tends to increase the amount of particle. Nevertheless, it was found that the effect of ratio of overfired air to total air supply is more pronounced, since decrease in this ratio, from 0.3 to 0.1, the amount of particles are decreased significantly.

(3) During cofiring fuel mixture at 70% of rice husk mass fraction, it is found that there are two major fraction of particle size; 40 < *D*_*p*_ < 70 nm to 0.2 < *D*_*p*_ < 0.5 *μ*m. This indicates the possibility of agglomeration of ultrafine particle increasing the average diameter of particle.

(4) The analysis of particle morphology shows that the isolate shape of submicron particle produced during lignite combustion is characterised by different geometries such as round, capsule, rod, flake-like, whereas the spherical shape is obtained with rice husk combustion. 

## Figures and Tables

**Table 1:** Fuel properties.

Fuel	Proximate analysis (% wt, as received)				Ultimate analysis (% wt, dry ash free)					HHV	
	Moisture	Volatile matter	Fixed carbon	Ash	C	H	O	N	S	(MJ/kg)	
Lignite	9.0	41.4	33.1	11.2	62.7	4.1	27.0	1.1	5.2	18.9	
Rice husk	8.5	57.5	17.2	16.8	49.1	6.3	44.4	0.2	—	14.1	
Bagasse	14.2	68.0	20.3	2.5	43.1	5.1	51.4	0.3	—	17.8	

	Oxide composition of fuel ash (% wt)										

	SiO_2_	Al_2_O_3_	Fe_2_O_3_	CaO	TiO_2_	MgO	SO_3_	P_2_O_5_	Na_2_O	MnO_2_	K_2_O

Lignite	34.38	16.50	12.80	13.70	0.36	1.87	17.05	0.16	0.90	0.11	2.72
Rice husk	92.70	0.14	2.00	0.54	0.02	0.35	0.37	0.43	0.07	0.19	2.50
Bagasse	42.90	23.80	16.90	2.20	2.50	2.10	0.60	1.30	0.60	ND	3.20

**Table 2:** Total number/mass concentration of particles.

Fuel	Fuel density (kg/m^3^)	Total number (particles/cm^3^ · kg_fuel_)	Total mass concentration (mg/Nm^3^ · kg_fuel_)
Lignite	736	3.4 × 10^3^	12.2
Rice husk	90	1.6 × 10^4^	8.0
Bagasse	60	1.5 × 10^5^	6.5

Total air flow rate: 300 LPM and OFA/TA: 0.3.

**Table 3:** Total number/mass concentration of particle.

Lignite/Rice husk mass fraction	OFA/TA	Fuel Density (kg/m^3^)	Burning Rate (g/sec) [12]	Total number (particles/cm^3^ · kg_fuel_)	Total mass concentration (mg/Nm^3^ · kg_fuel_)
30/70	0.3	120	0.44	2.2 × 10^5^	20.5
60/40	0.3	220	0.42	6.3 × 10^4^	15.3
60/40	0.1	220	0.57	8.7 × 10^3^	2.1

Total air flow rate: 300 LPM.
